# Pandemic Influenza and Excess Intensive-Care Workload

**DOI:** 10.3201/eid1410.080440

**Published:** 2008-10

**Authors:** Raoul E. Nap, Maarten P.H.M. Andriessen, Nico E.L. Meessen, Dinis dos Reis Miranda, Tjip S. van der Werf

**Affiliations:** University of Groningen, Groningen, the Netherlands

**Keywords:** pandemic, intensive care, healthcare workers, preparedness, planning, research

## Abstract

In the Netherlands a major part of preparedness planning for an epidemic or pandemic consists of maintaining essential public services, e.g., by the police, fire departments, army personnel, and healthcare workers. We provide estimates for peak demand for healthcare workers, factoring in healthcare worker absenteeism and using estimates from published epidemiologic models on the expected evolution of pandemic influenza in relation to the impact on peak surge capacity of healthcare facilities and intensive care units (ICUs). Using various published scenarios, we estimate their effect in increasing the availability of healthcare workers for duty during a pandemic. We show that even during the peak of the pandemic, all patients requiring hospital and ICU admission can be served, including those who have non–influenza-related conditions. For this rigorous task differentiation, clear hierarchical management, unambiguous communication, and discipline are essential and we recommend informing and training non-ICU healthcare workers for duties in the ICU.

## Abstract 2

Even during the peak of a pandemic, all patients requiring hospital and ICU admission can be served, including those who have non–influenza-related conditions.

In the Netherlands a major part of preparedness planning for an epidemic or pandemic, e.g., avian influenza A, consists of maintaining essential services provided by the police, fire departments, army personnel, and healthcare workers (HCWs). Even if an effective vaccine against avian influenza (H5N1) would be available (*1*), preparation for a pandemic is still vital to maintain optimal care for acute-care patients and those with influenza-like illness (ILI). The preparation for excess workloads among HCWs becomes even more important with the emergence of highly pathogenic avian influenza strains.

We present a model to show the impact of the increased demand in HCWs with the increase in the number of hospitalized patients. We factor in the notion that the number of HCWs will be reduced because of increased absenteeism, which in turn affects the utilization of intensive-care beds and mechanical ventilation capacity. We present scenarios aiding in temporarily increasing the work force of HCWs in the intensive-care unit (ICU) environment using different additive strategies. Because the surge capacity of intensive-care resources is typically limited (*2*), we explore what training and preparation HCWs and managers at different levels will need to face the challenges posed by pandemic influenza.

## Methods

### Setting

The University Medical Center Groningen (UMCG) is a tertiary-care university hospital covering ≈12% of the total Dutch population and ≈30% of the total surface area of the Netherlands. Under Dutch law, UMCG plays a dominant role in the region to organize and coordinate healthcare surge capacity during a catastrophe such as an avian influenza pandemic. With regional and municipal health authorities, general practitioners, and medical and managerial representatives of all 15 hospitals in the northern Netherlands region, training courses were organized for pandemic influenza. These courses emphasized the need for enhanced collaboration, sharing of information, and communication. Part of this training course was the development of an epidemiologic model to access the regional impact of a pandemic and the extent of possible preparations (*3*) at both managerial and medical domains.

### Basic Model

We used FluSurge 2.0 (*4*) and a computer model in an Excel (Microsoft, Redmond, WA, USA) file developed by one of the authors to calculate the impact of an influenza pandemic in the Netherlands on hospital admission and occupancy rate of all ICU beds (i.e., those with facilities for mechanical ventilation) (*3*,*5*). Data on population (slightly >1.7 million) and age distribution were obtained from publicly available sources. Because age distribution in the Dutch population data was provided in blocks of 5 years, we converted these data to an even distribution to enable calculations with the FluSurge program (*6*). Data on total hospital beds, ICU beds, and number of nurses and their fulltime equivalents were obtained from publicly available sources (*7*). Information on ICU capacity was also obtained from reports from hospital administrators during the training sessions. These data on reported ICU capacity were discussed during a semistructured telephone interview with intensive-care specialists (usually anesthetists or internists) in August 2006. On the basis of these data, we estimated the regular bed capacity and maximal surge capacity. Numbers on the effects of pandemic influenza on healthcare services were adopted from the National Institute for Public Health and the Environment (RIVM) (*5*,*8*). RIVM presented tables for 25% and 50% disease attack rates (ARs) that represented best and worst case scenarios. From these tables, we calculated the 30% AR by linear transformation. A 30% AR is the most likely scenario according to the Centers for Disease Control and Prevention and RIVM. The AR was defined as the percentage of the population that became ill.

We also calculated within the model the total number of patients admitted to the hospitals at each point in time during the pandemic. We defined the first day (day 0) as the moment the World Health Organization (WHO) declares effective human-to-human transmission (phase IV or V in the current WHO phase of pandemic alert). We assume the pandemic would reach the Netherlands ≈15 days after this first confirmed, effective human-to-human transmission.

We took into account the time each patient would occupy a hospital or ICU bed (range 8–15 days), based on experience with patients admitted to ICU with diagnoses of pneumonia or sepsis. Finally, we incorporated estimated risk for death per patient, a rate that in turn would reduce the number of admitted patients at any 1 time. Because RIVM data are in weekly blocks, we evenly distributed the number of hospital admissions and deaths during weekdays. We also factored in our calculations the effect of treatment (within 48 hours of infection) with antiviral medication on the spread and impact of the pandemic, although the exact effect size is still uncertain (*6*,*9*). Antiviral medication is assumed to reduce the total number of hospital admissions by 50% and death by ≈30%.

In the basic model we incorporated the probable absenteeism of HCWs attributable to illness. We assumed that HCWs will become ill at a similar rate as the normal population. We used the total number of HCWs and linearly transformed the ratios of illness and death of the population over time on the HCW database. We focused our preparedness plan on adults and assumed a pattern in the outbreak similar to that for Spanish flu (*10*) and severe acute respiratory syndrome, in which adolescents and adults accounted for most patients (Technical Appendix).

### Modeling Nursing Workload

We developed an extension to this model that showed the effects of increased demand in the HCW force because of an increased number of hospitalized patients with ILI. In this model, we factored in the notion that the number of available key HCWs (e.g., physicians, intensive-care nurses) would be reduced because of ILI absenteeism, which affects the use of ICU beds and mechanical ventilation capacity.

Furthermore, using different additive strategies reported in the literature, we present several alternatives to increase the number of available HCWs. All 3 scenarios were adopted because of their inherent ease of use in daily hospital practice.

We used the basic model (*3*,*5*) for peak hospital and ICU occupancy rates during a pandemic of influenza. A major part of preparation encompasses clearing all 15 regional hospitals of general surgical and medical patients, leaving ≈30% of acute-care capacity for non–influenza-related illness. In these 15 hospitals, the total ICU capacity is 200 beds. The predominant use of ICU beds in Europe is for postoperative care (instead of recovery rooms or postanesthesia special-care units) (*11*). Of these, 60 ICU beds (30%) need to be reserved for acute care for non–influenza-related illness, leaving ≈140 ICU beds for influenza-related patients. The mean ICU length of stay for postoperative and general medical care patients is 3.4 (median 1 day, standard deviation [SD] 7.4) days. Therefore, it follows that ≈96% of these 140 ICU beds occupied by postoperative and general medical care patients at the beginning of the pandemic can be made available within a 14-day period for patients ill from flu.

Even though we assume HCWs will become ill at the same rate as the general population, we also assume that morale and professionalism will be high and that undue absenteeism will not occur. That is, adherence to professional standards of HCWs reporting for duty will be high, although some unavailability of HCWs might be expected because of care duties within families or among neighbors who may become ill (*12*). This erosion has not been taken into account in the model we present.

To model the available number of HCWs in relation to the maximum number needed in a pandemic, we translated the peak ICU occupancy rate into a severity of illness and workload scoring system. A variety of validated scoring systems for patient severity and workload exist for use in the ICU environment and are widely used (*13*–*18*).

We used data derived from the databases of the European Research in Intensive Care Units (EURICUS) projects, a multicenter project designed to study the ICUs of the European Union (EU), to project severity of illness, diagnosis, and workload data in the ICU. A description of the methods, objectives, and results of these projects have been reported (*11*,*15*,*19*,*20*). These projects were developed from 1994 through 2000 and included 137 ICUs from 14 different EU states with a total of 43,027 individual patient records and 227,209 nursing days. Many study variables were analyzed, including age, diagnosis, length of stay, Simplified Acute Physiology Score (SAPS-II) (*14*), Sequential Organ Dysfunction Score (SOFA-II ) (*21*), ICU mortality rate, hospital mortality rate, and Glasgow Coma Score. Part of this research was the measurement of nursing workload in ICUs (*15*–*17*). Nursing workload per intensive-care patient was calculated by using the Nine Equivalents of Nursing Manpower use score (NEMS) (*16*). NEMS is a therapeutic index to measure nursing workload at the ICU level. The use of NEMS has been developed and validated for multicenter ICU studies; management purposes in the general (macro) evaluation and comparison of workload at the ICU level; and prediction of workload and planning of nursing staff allocation at the individual patient level. NEMS correlates highly with all currently used severity of illness scoring systems such as Acute Physiology, Age, Chronic Health Evaluation III, SAPS-II, and SOFA-II (*16*,*20*). The major advantage of all these scoring systems is that they are an objective and reproducible measure of nursing workload related to the various activities performed in the ICU, without considering the appropriateness of standing policies of medical care.

We attributed scores on the basis of a specific diagnostic category (*13*) to the corresponding NEMS points from the EURICUS projects database. We used the medical diagnostic category “bacterial/viral pneumonia” (diagnosis category 15) as an indicator for nursing care requirements of ILI patients, as this closely matches the medical condition typical for influenza patients. Furthermore, we used the average NEMS points for all patients (minus the patients with diagnosis 15) in the EURICUS database as an indicator for the nursing workload necessary for the non-ILI acute-care patients. We modeled the available HCWs with a 30% AR, 25% and 50% ICU admission rate, and mean length of stay of 8 days and 15 days without antiviral medication (AVM) (pandemic period 9 weeks) and 30% AR, 25% and 50% ICU admission rate, and mean length of stay of 8 days or 15 days with AVM (pandemic period 14 weeks).

### Scenarios

#### Scenario 1

Increasing the number of HCWs by expanding the work shift from 8 to 12 hours would increase the number of operational HCWs by ≈50%. The huge strain on personnel is justified, when one considers the relatively short peak surge period, and we expect HCWs to comply.

#### Scenario 2

HCWs will become sick with ILI at the same rate as the general population. Lee and Chen (*22*) showed that 8 weeks’ prophylactic use of neuraminidase inhibitors decreased peak absenteeism among HCWs from 10% to 2%. We translated this in our model by reducing the number of lost NEMS points (e.g., number of lost HCWs) by 80% for the entire pandemic period.

#### Scenario 3

In an international ICU workload study, Miranda et al. (*17*) showed that tasks performed in normal ICU environment by ICU nurses are only 30% dedicated to technical and specific ICU tasks for which ICU nurses are trained. This result means 70% of all tasks in ICU are regular nursing tasks performed daily in non-ICU, standard healthcare hospital environments. In this scenario, we mathematically decreased the number of NEMS points to be covered by ICU nurses to 30%. Non-ICU nurses and other hospital personnel (communication advisors, psychologists, physicians, administrative staff, and the like) have to be prepared and trained to take over, for a relatively short period, the 70% of nontechnical duties regularly performed by ICU nurses. We assume that there will be substantial numbers of HCWs in wards and ancillary facilities available because all hospitals involved will stop admitting postoperative care and non–influenza-related medical patients when WHO declares the pandemic alert. This will free ≈70% of all HCWs in the 15 hospitals.

#### Scenario 4

Finally, we combined the effects of all 3 scenarios in an overall model of available HCWs. This final model gives the opportunity to study the effect of 3 easy-to-implement scenarios.

## Results

The Table shows the result of our baseline calculations. For the 30% non-ILI acute-care patients, we attributed 24.8 (SD 9.9) mean NEMS points; for the ILI patients, we attributed 28.6 (SD 9.8) mean NEMS points. We assume that an ICU nurse can deliver 46.3 NEMS points in 24 hours. Because HCWs will become ill as the population does, the number of NEMS points available will be reduced as the pandemic period progresses; the lowest number will occur at 28 days, when the pandemic period is assumed to be at its highest point (Appendix Table 1). The lowest number of NEMS points will occur at day 43, when AVM is available for the population (Appendix Table 2). As can be seen from both (Appendix Tables 1, 2) HCW shortage will occur if the ICU admission rate increases to 50% (mean length of stay 8 days or 15 days without AVM). However, if AVM is available, no HCW shortage will occur, under the premises that all general medicine and surgery have been temporarily stopped.

**Table:** Study population characteristics from EURICUS projects*

Characteristic	Study population	Diagnosis category 15†
Total no.	39,158	1,413
ICU length of stay, mean (SD)	5.2 (10.4)	10.5 (13)
Age, no. (%)	59.3 (19.8)	57.7 (21.8)
SAPS-II, no. (%)	31.9 (17.8)	39.5 (18.8)
NEMS, no. (%)	24.8 (9.9)	28.6 (9.8)
ICU deaths, %	13	27
Overall deaths, %	19	36

*ICU, intensive care unit; SAPS, Simplified Acute Physiology Score; NEMS, Nine Equivalents of Nursing Manpower use score. †Values are given as mean (SD).

### Scenarios

#### Scenario 1

**Figure 1:**
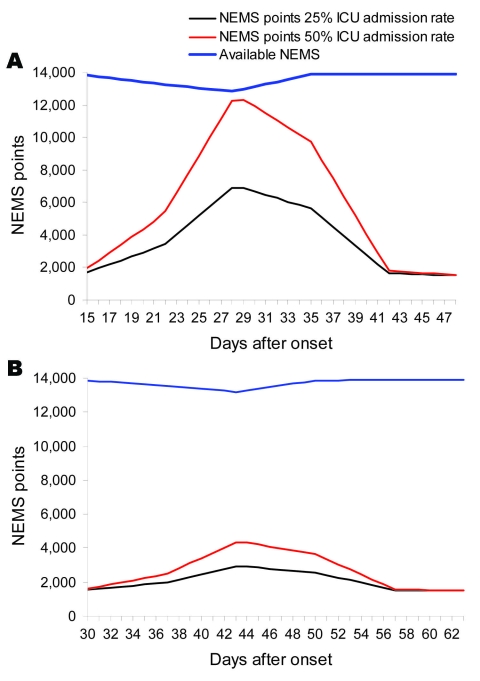
A) Amount of Nine Equivalents of Nursing Manpower use (NEMS) points needed and available by 25% and 50% admission rate in the intensive care unit (ICU) scenario 1, expanding healthcare worker (HCW) work hours from 8 to 12 h per 24 h (pandemic period 9 wk). B) Amount of NEMS points needed and available by 25% and 50% admission rate in ICU scenario 1, expanding HCW work hours from 8 to 12 h per 24 h (pandemic period 14 wk).

In Figure 1, panels **A** and **B**, the results are given for the model length of stay of 15 days, with 25% and 50% ICU admission rates with and without AVM. Even in the worst case scenario, a 15-day length of stay without availability of AVM for the individual patient (Figure 1, panel **A**), no staff shortage appears. The models for a length of stay of 8 days, 25% and 50% ICU admission with and without AVM (not shown), show no staff shortage over time.

#### Scenario 2

**Figure 2:**
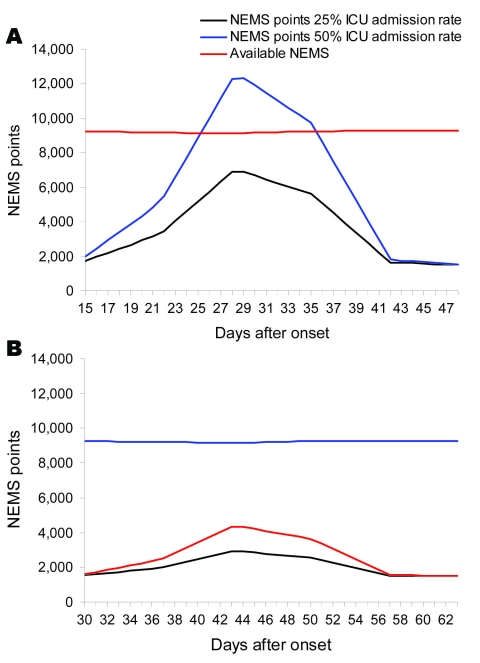
A) Amount of Nine Equivalents of Nursing Manpower use (NEMS) points needed and available by 25% and 50% admission rate in the intensive care unit (ICU) scenario 2, healthcare worker (HCW) 8 wk prophylactic use of neuraminidase inhibitors (pandemic period 9 wk). B) Amount of NEMS points needed and available by 25% and 50% admission rate in the ICU scenario 2, HCW 8 wk prophylactic use of neuraminidase inhibitors (pandemic period 14 wk).

If the number of HCWs who become ill from ILI is reduced by 80%, a similar increase of deliverable NEMS points is achieved. This increase is irrespective of the number of patients admitted to the hospital and ICU. In Figure 2, panels **A** and **B**, results are given for the models with a 15-day length of stay, 25% and 50% ICU admission rate, with and without AVM. In this model, the effect of potential HCW staff shortage is most profound at a 50% ICU admission rate. Even decreasing the number of ill HCWs because of 8 weeks’ prophylactic use of neuraminidase inhibitors (*22*) does not solve staff shortage when it is most needed.

#### Scenario 3

**Figure 3:**
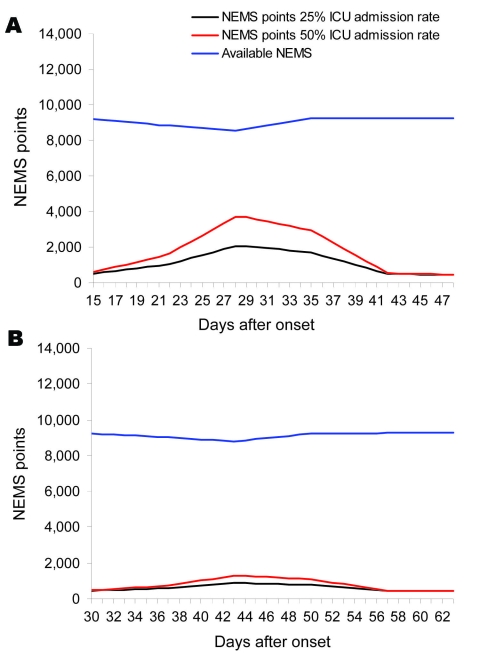
A) Amount of Nine Equivalents of Nursing Manpower use (NEMS) points needed and available by 25% and 50% admission rate in intensive-care unit (ICU) scenario 3, task differentiation in healthcare workers (HCWs) (pandemic period 9 wk). B) Amount of NEMS points needed and available by 25% and 50% admission rate in ICU scenario 3, task differentiation in HCWs (pandemic period 14 wk).

Transfer of tasks to other HCWs than ICU nurses decreases the number of NEMS points needed by 70%. We assume acute-care and ILI patients admitted to the ICU will require the 30% technical ICU-related work of ICU nurses. This decrease in the number of NEMS points needed has a direct effect on the necessary workload in the different AR and length-of-stay models. Figure 3, panels **A** and **B**, shows that decreasing the number of required NEMS in the ICU results in sufficient numbers of HCWs being available for care of ILI patients.

#### Scenario 4

**Figure 4:**
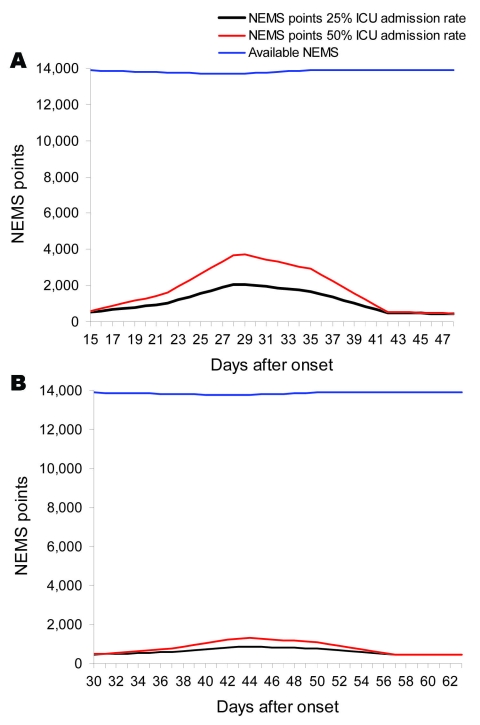
A) Amount of Nine Equivalents of Nursing Manpower use (NEMS) points needed and available by 25% and 50% admission rate in intensive care unit (ICU) scenarios 1, 2, and 3, combined effect of all 3 scenarios (pandemic period 9 wk). B: Amount of NEMS points needed and available by 25% and 50% admission rate in ICU scenario 1,2, and 3, combined effect of all 3 scenarios (pandemic period 14 wk)

We combined all 3 scenarios to access the impact of the 3 relatively easy to implement scenarios on HCW availability. Figure 4, panels **A** and **B**, shows the effects of all 3 scenarios on the NEMS points availability.

## Discussion

We provide calculations, which incorporate HCW absenteeism, for the surge capacity in HCWs in case of an influenza pandemic. In our model, we have shown that business continuity is maintainable when strict, clear, and disciplined hierarchical structures are in place.

### Recommendation 1

Start preparations in time. When WHO declares phase IV, stop admitting all general patients, clear hospital beds, and free up HCWs. If hospitals are not cleared from responsibility for postoperative and general medical patients and if admitting general patients is not stopped, not only will there not be enough hospital and ICU beds available for ILI patients but also unnecessary risk for exposure to ILI will be placed on these patients. This step is mandatory in all hospital preparedness planning.

### Recommendation 2

Implement a simple nursing workload measurement system for scarce resources. This will provide valuable information on the current workload and will aid in planning for a pandemic (*23*–*26*).

### Recommendation 3

Gain insight, per hospital, about general and acute-care populations. This will provide information about the number of hospital and ICU beds and the number of HCWs capable of being cleared for work with ILI patients.

Length of stay for individual ICU patients is the predominant factor in capacity planning for a pandemic. We show in our results, for a worst case scenario of patients kept in ICU for 15 days, that demand for workload and ICU beds exceeds capacity (*3*). Reducing the length of stay of some patients will increase the capability of hospitals to serve most ILI patients. Therefore, we propose that a strong distinction be made between cure and care. During a pandemic, elderly patients with severe co-existing conditions may opt for supportive care without hospital and/or ICU admission, in consultation with their loved ones and their general practitioners. These patients may die because of ILI.

The smallest gain in available NEMS points occurred with scenario 2, i.e., 8 weeks’ prophylactic use of neuraminidase inhibitors for HCWs. Although prophylactic use of neuraminidase inhibitors serves an import role in staff protection (*27*) and will most likely enhance HCW compliance, it serves its role specifically at the individual HCW level instead of at the workload level. The largest effect on availability of NEMS points is with scenario 3. Through insight about workload of ICU nurses and other HCWs, rigorous task differentiation can be obtained, and even specific tasks can be delegated to non-HCW specialists (for example, communication with family members of deceased patients can be done by hospital spokespersons and communications experts).

Furthermore, if all general medical and surgical patients are cleared from all hospitals in the northern part of the Netherlands, 932 medical specialists (anesthesiologists, surgeons, internal medicine, cardiologists, and cardiothoracic surgeons) can be used to provide care for acute-care and influenza patients. This strategy would greatly enhance the number of HCWs for pandemic influenza per hospital.

Finally, UMCG has 2,470 undergraduate and graduate medical students, 367 dentistry students, and 423 students’ behavioral and social sciences in 2006. There are also 1,240 nursing science students and 2,391 health science students at the Hanze University Groningen of Applied Sciences. If indeed 44.3% (*28*) (226 of 510 medical students) of healthcare-related students were to report for duty in case of a HCW shortage, 3,053 extra HCWs could be recruited for duty during a pandemic to fill the potential gaps in healthcare delivery. These students would be distributed among all 15 hospitals in the region, giving each ≈203 extra HCWs.

There are several limitations to our analysis. The report by the Writing Committee of the Second World Health Organization Consultation on Clinical Aspects of Human Infection with Avian Influenza (*29*) prompts us to discuss the impact these findings have on preparedness planning of healthcare organizations. The case-fatality rate of 61% in total, especially among persons 10 to 19 years of age, and the much lower rate in persons >50 years of age are different from the reported rates of past pandemics (*30*–*32*). This increased case-fatality rate in the 10- to 19-year age group will have a tremendous effect on pandemic preparedness planning and the model presented here for hospital and healthcare institutions. Until now, we assumed persons 10–19 years of age made up only a small percentage of potential hospitalized persons (*3*). The question that has not been answered by the Writing Committee is whether this age group’s particular risk is because this age group is mainly responsible for handling poultry and poultry products in reports on avian influenza. In the Netherlands, there are ≈2 million persons (12%) in this age category among the country’s 16.4 million inhabitants. The same proportion holds for the 27 countries of the European Union (494 million inhabitants, 57 million persons 10–19 years of age [11.7%]).

In affluent countries, preparation for a pandemic is mostly supported and financed at the national level, and the overall belief is that hospital and intensive-care capacity will suffice. Less affluent countries might have more difficulties with pandemic preparation. The Writing Committee does not provide guidelines for preparation. For affluent countries, the economic ramifications of “losing” the younger generation struck by avian influenza might be even more dramatic then a pandemic itself because affluent countries are dealing with an increasing older population and lower birth rates.

The model used also has several limitations. The datasets of the EURICUS projects were constructed almost a decade ago. Because of changes in ICU technology and ICU nursing, decreasing emphasis on technical procedures, increased emphasis on communication within the ICU team, and communication with the patients’ relatives and loved ones, some variables like NEMS (change in workload), length of stay, and ICU mortality rate (change in technical procedures) may have changed. We expect that our model presents a worst-case scenario for workload. In addition, our basic model is based on incomplete and sometimes conflicting or inconsistent information on the effects of an influenza pandemic. We assume that more reliable data will only become available in the early stages of a pandemic.

Rigorous task differentiation, clear hierarchical management, unambiguous communication, and discipline are essential. We recommend informing and training non-ICU HCWs for possible duties at the ICU. Training should address HCW needs as well as those of family and loved ones of patients with ILI admitted to the ICU. It should also incorporate the potential difficulties for HCWs in communicating with family members and loved ones if patients die after intensified treatment decisions (*3*,*33*,*34*).

## Supplementary Material

Appendix Table 1 Total NEMS points needed for ICU surge capacity and difference with HCWs available, ICU length of stay of 8 d or of 15 d without antiviral medication, ICU admission rate of 25% and 50% inclusive of acute-care demand*

Appendix Table 2 Total NEMS points needed for ICU surge capacity and difference with HCW available NEMS points, ICU length of stay 8d and 15d with antiviral medication, ICU admission rate 25% and 50% inclusive of acute-care demand*

Technical Appendix Models for estimating health care demands, incidence and prevalence in different scenarios and intervention strategies
