# Supplementary material for: Pandemic Influenza and Excess Intensive-Care Workload
Source: Emerg Infect Dis. 2008 Oct;14(10):1518–25. doi: 10.3201/eid1410.080440 (PMC2609860; doi:10.3201/eid1410.080440)
Supplement: Appendix Table 1 — Total NEMS points needed for ICU surge capacity and difference with HCWs available, ICU length of stay of 8 d or of 15 d without antiviral medication, ICU admission rate of 25% and 50% inclusive of acute-care demand* [file 08-0440_appT1-s1.pdf]

Publisher: CDC; Journal: Emerging Infectious Diseases
Article Type: Research; Volume: 14; Issue: 10; Year: 2008; Article ID: 08-0440
DOI: 10.3201/eid14/10/080440; TOC Head:

Appendix Table 1. Total NEMS points needed for ICU surge capacity and difference with HCWs available, ICU length of stay of 8 d or of 15 d without antiviral medication, ICU admission rate of 25% and 50% inclusive of acute-care demand*

	Mean ICU length of stay 8 d				Mean ICU length of stay 15 d			
	ICU admission rate 25%		ICU admission rate 50%		ICU admission rate 25%		ICU admission rate 50%	
Days after onset	NEMS points needed	Difference between points needed and available	NEMS points needed	Difference between points needed and available	NEMS points needed	Difference between points needed and available	NEMS points needed	Difference between points needed and available
15	1,724	7,481	1,960	7,245	1,717	7,488	1,974	7,230
16	1,960	7,189	2,432	6,718	1,974	7,175	2,432	6,718
17	2,196	6,898	2,904	6,190	2,203	6,891	2,918	6,176
18	2,432	6,607	3,376	5,663	2,432	6,607	3,376	5,663
19	2,668	6,316	3,848	5,136	2,661	6,323	3,862	5,122
20	2,904	6,024	4,319	4,609	2,918	6,010	4,319	4,609
21	3,140	5,733	4,791	4,082	3,147	5,726	4,806	4,067
22	3,472	5,354	5,456	3,370	3,461	5,364	5,463	3,362
23	3,805	4,974	6,121	2,658	4,033	4,745	6,607	2,171
24	4,137	4,595	6,786	1,946	4,605	4,126	7,723	1,009
25	4,470	4,215	7,451	1,234	5,177	3,507	8,867	–182
26	4,802	3,836	8,116	522	5,749	2,888	10,011	–1,373
27	5,135	3,456	8,781	–190	6,321	2,269	11,155	–2,564
28	5,467	3,077	9,446	–902	6,893	1,650	12,270	–3,726
29	4,920	3,723	8,352	291	6,893	1,750	12,327	–3,684
30	4,373	4,369	7,258	1,484	6,693	2,049	11,898	–3,156
31	3,826	5,016	6,164	2,678	6,464	2,377	11,469	–2,628
32	3,279	5,662	5,070	3,871	6,264	2,677	11,040	–2,099
33	2,732	6,308	3,976	5,064	6,035	3,005	10,611	–1,571
34	2,185	6,955	2,882	6,258	5,835	3,305	10,182	–1,043
35	1,638	7,601	1,788	7,451	5,606	3,633	9,753	–514
36	1,617	7,625	1,745	7,497	5,063	4,179	8,609	633
37	1,595	7,650	1,703	7,543	4,491	4,754	7,465	1,780
38	1,574	7,674	1,660	7,588	3,919	5,329	6,350	2,898
39	1,552	7,699	1,617	7,634	3,347	5,904	5,206	4,045
40	1,531	7,723	1,574	7,680	2,775	6,479	4,062	5,192
41	1,509	7,748	1,531	7,726	2,203	7,054	2,918	6,339
42	1,488	7,772	1,488	7,772	1,631	7,629	1,803	7,457
43	1,488	7,772	1,488	7,772	1,631	7,629	1,745	7,515

*NEMS, Nine Equivalents of Nursing Manpower use score; HCWs, healthcare workers; ICU, intensive care unit.
