# Supplementary material for: Pandemic Influenza and Excess Intensive-Care Workload
Source: Emerg Infect Dis. 2008 Oct;14(10):1518–25. doi: 10.3201/eid1410.080440 (PMC2609860; doi:10.3201/eid1410.080440)
Supplement: Appendix Table 2 — Total NEMS points needed for ICU surge capacity and difference with HCW available NEMS points, ICU length of stay 8d and 15d with antiviral medication, ICU admission rate 25% and 50% inclusive of acute-care demand* [file 08-0440_appT2-s2.pdf]

Publisher: CDC; Journal: Emerging Infectious Diseases
Article Type: Research; Volume: 14; Issue: 10; Year: 2008; Article ID: 08-0440
DOI: 10.3201/eid14/10/080440; TOC Head:

Appendix Table 2. Total NEMS points needed for ICU surge capacity and difference with HCW available NEMS points, ICU length of stay 8 d and 15 d with antiviral medication, ICU admission rate 25% and 50% inclusive of acute-care demand*

	Mean ICU length of stay 8 d				Mean ICU length of stay 15 d			
	ICU admission rate 25%		ICU admission rate 50%		ICU admission rate 25%		ICU admission rate 50%	
Days after onset	NEMS points needed	Difference between points needed and available	NEMS points needed	Difference between points needed and available	NEMS points needed	Difference between points needed and available	NEMS points needed	Difference between points needed and available
30	1,550	7,680	1,612	7,618	1,550	7,680	1,612	7,618
31	1,612	7,588	1,736	7,464	1,612	7,588	1,736	7,464
32	1,674	7,496	1,860	7,311	1,674	7,496	1,860	7,311
33	1,736	7,405	1,983	7,157	1,736	7,405	1,983	7,157
34	1,798	7,313	2,107	7,003	1,798	7,313	2,107	7,003
35	1,860	7,221	2,231	6,849	1,860	7,221	2,231	6,849
36	1,921	7,129	2,355	6,696	1,921	7,129	2,355	6,696
37	2,008	7,005	2,529	6,484	2,008	7,005	2,529	6,484
38	2,095	6,880	2,702	6,273	2,157	6,818	2,826	6,149
39	2,182	6,756	2,876	6,062	2,306	6,632	3,123	5,814
40	2,269	6,631	3,049	5,850	2,454	6,445	3,421	5,479
41	2,356	6,507	3,223	5,639	2,603	6,259	3,718	5,144
42	2,442	6,382	3,397	5,428	2,752	6,073	4,016	4,809
43	2,529	6,258	3,570	5,217	2,901	5,886	4,313	4,474
44	2,386	6,464	3,284	5,566	2,906	5,944	4,324	4,526
45	2,243	6,671	2,997	5,916	2,850	6,063	4,211	4,702
46	2,099	6,877	2,711	6,265	2,793	6,183	4,099	4,878
47	1,956	7,083	2,424	6,615	2,737	6,303	3,986	5,054
48	1,813	7,290	2,138	6,965	2,680	6,422	3,873	5,230
49	1,670	7,496	1,851	7,314	2,624	6,542	3,760	5,406
50	1,526	7,702	1,565	7,664	2,568	6,661	3,647	5,582
51	1,521	7,712	1,554	7,679	2,419	6,814	3,350	5,884
52	1,515	7,722	1,543	7,695	2,270	6,968	3,052	6,185
53	1,510	7,732	1,532	7,710	2,121	7,121	2,755	6,487
54	1,504	7,742	1,521	7,726	1,973	7,274	2,457	6,789
55	1,499	7,752	1,510	7,741	1,824	7,427	2,160	7,091
56	1,493	7,762	1,499	7,757	1,675	7,580	1,862	7,393
57	1,488	7,772	1,488	7,772	1,526	7,734	1,565	7,695
58	1,488	7,772	1,488	7,772	1,521	7,739	1,554	7,706

*NEMS, Nine Equivalents of Nursing Manpower use score; HCWs, healthcare workers; ICU, intensive care unit.
