# Supplementary material for: Pandemic Influenza and Excess Intensive-Care Workload
Source: Emerg Infect Dis. 2008 Oct;14(10):1518–25. doi: 10.3201/eid1410.080440 (PMC2609860; doi:10.3201/eid1410.080440)
Supplement: Technical Appendix — Models for estimating health care demands, incidence and prevalence in different scenarios and intervention strategies [file 08-0440_Techapp-s3.pdf]

Pandemic Influenza and Excess Intensive-Care Workload

Technical Appendix

Models for estimating health care demands, incidence and prevalence in different scenarios and intervention strategies

In the models the following assumptions were made:

- Attack rates of 25%, 30% and 50%;
- The age specific attack and complication rates are as in a normal influenza epidemic;
- Health care including application of antibiotics will be equal to a normal influenza epidemic;
- Therapeutic use of one treatment of neuraminidase inhibitors (applied within 48 hours after onset of symptoms) gives 50% reduction in hospital admissions and mortality;
- No upper limit inhibitors shortage has been incorporated in models;
- Total high risk group per 100,000 inhabitants is based on registrations from the general practitioners databases;
- Duration of the pandemic period is based on historical data, although local and regional differences in duration can occur;
- Basic reproductive number R_0 was set on 1.4

Formulae (adapted from Hagenaars et al (*1*) and Van Genugten et al (*2*))
TotPop = Total Population divided in age and risk groups
PopatRisk = Population at risk
HCcmr Influenza-like illness = Number of general practitioner consults per 100,000 inhabitants
ZHObaltussen = Number of hospital admissions per 100,000 inhabitants (adapted from Baltussen(*3*))
Ssprenger = Mortality contributable to influenza per 100,000 inhabitants (adapted from Sprenger(*4*))
HCrate = General practitioners consultation rate for influenza-like illness
ZHOrate = Hospital admission rate for influenza
Srate = Mortality rate as a result from influenza
AR_Pandemic / Normal Epidemic = Rate attack rates pandemic versus ‘normal’ epidemic

Formulae ‘non-intervention scenario’
HCrate = HCcmr Influenza-like illness
ZHOrate = ZHObaltussen
Srate = Ssprenger
PopatRisk = TotPop

Number of general practitioner consultations = HCrate * PopatRisk * AR_Pandemic / NormaleEpidemic
Number of Hospital admission = ZHOrate * PopatRisk * AR_Pandemic / Normal Epidemic
Mortality = Srate * PopatRisk * AR_Pandemic / NormaleEpidemic

Table 1. Input values for the model:

A) High risk proportion of the population for the three Northern provinces of the Netherlands.

Age groups, y	Low risk proportion of the population	High risk proportion of the population*
0–18	97.6%	2.4%
19–64	93.8%	6.2%
≥65	65.0%	35.0%

*High risk proportion of the population consists of a number of diseases identified as contributors to influenza-related excess mortality. These include pneumonia, cerebral-vascular accident, chronic heart disease and diabetes mellitus (*3*).

B) Age specific attack rates (*2*) by age group and 30% attack rate.

Age groups, y	Attack rate, %
0–18	37.4
19–64	28.6
≥65	23.1

C) Death rates (*4*) per 100,000 population by age and risk group and 30% attack rate

Age groups, y	Low risk population	High risk population
0–18	1.83	89.25
19–64	1.83	89.25
≥65	78.72	254.76

D) Hospitalization rates (*3*) per 100,000 population by age and risk group and 30% attack rate.

Age groups, y	Low risk population	High risk population
0–18	1.2	300
19–64	1.2	300
≥65	120	555

E) Absolute number of outpatient visits† (*2*) by 30% attack rate for the three Northern provinces in the Netherlands.

Age groups, y	No. outpatient visits
0–18	36,921
19–64	72,044
≥65	12,572
**Total**	**121,537**

†Outpatient visits were set to zero in our model. Part of the preparedness plan encompasses that outpatient visits will be covered by GPs. GPs in the region have trained and prepared for this task.

F) Avian influenza impact for the three Northern provinces in the Netherlands, 25% attack rate and pandemic period 9 weeks

Week	Days	No. patients	General practitioner consultations	Hospital admissions	Deaths
0	1–7	0	0	0	0
1	8–14	85	9	0	0
2	15–21	3,811	418	0	0
3	22–28	118,198	13,415	255	17
4	29–35	281,381	36,216	800	340
5	36–42	21,013	2,994	68	51
6	43–49	459	67	0	0
7	50–56	17	0	0	0
8	57–63	17	0	0	0
9	64–70	0	0	0	0
**Total**		**424,981**	**53,119**	**1,123**	**408**

G) Avian influenza impact for the three Northern provinces in the Netherlands, 30% attack rate and pandemic period 9 weeks

Week	Days	No. patients	General practitioner consultations	Hospital admissions	Deaths
0	1–7	0	0	0	0
1	8–14	105	11	0	0
2	15–21	4,694	515	11	0
3	22–28	145,898	16,559	315	84
4	29–35	347,288	44,699	977	420
5	36–42	25,935	3,696	95	74
6	43–49	578	84	0	0
7	50–56	11	0	0	0
8	57–63	0	0	0	0
9	64–70	0	0	0	0
**Total**		**524,507**	**65,562**	**1,397**	**578**

H) Avian influenza impact for the three Northern provinces in the Netherlands, **50%** attack rate and pandemic period 9 weeks

Week	Days	No. patients	General practitioner consultations	Hospital admissions	Deaths
0	1–7	0	0	0	0
1	8–14	170	18	0	0
2	15–21	7,605	834	17	0
3	22–28	236,412	26,832	510	136
4	29–35	562,744	72,430	1,582	681
5	36–42	42,025	5,989	153	119
6	43–49	936	136	0	0
7	50–56	17	0	0	0
8	57–63	0	0	0	0
9	64–70	0	0	0	0
**Total**		**849,909**	**106,239**	**2,262**	**936**

Table 2A. estimated peak hospital occupancy rate related to mean length of stay range 8 to 15 days for 25, 30 and 50% attack rates and pandemic period 9 weeks, **without** antiviral medication

Mean length of stay, d	Attack rate, %		
	25	30	50
8	459	557	902
9	493	590	955
10	527	623	1,009
11	561	656	1,062
12	595	689	1,116
13	630	722	1,169
14	664	755	1,223
15	666	758	1,227

Table 2B. Estimated peak critical care occupancy rate by **25%** critical care admission rate*, related to mean length of stay range 8 to 15 days for 25, 30 and 50% attack rates and pandemic period 9 weeks, **without** antiviral medication

Mean length of stay, d	Attack rate, %		
	25	30	50
8	115	139	225
9	123	147	239
10	132	156	252
11	140	164	266
12	149	172	279
13	157	180	292
14	166	189	306
15	166	189	307

*Critical care admission rate, number of persons admitted to hospital with influenza likely to require admission to a critical care unit (% based on number of extra hospital admissions) (*5*)

Table 2C. Estimated peak critical care occupancy rate by **50%** critical care admission rate, related to mean length of stay range 8 to 15 days for 25, 30 and 50% attack rates and pandemic period 9 weeks, **without** antiviral medication

Mean length of stay, d	Attack rate, %		
	25	30	50
8	230	278	451
9	247	295	478
10	264	311	504
11	281	328	531
12	298	344	558
13	315	361	585
14	332	377	611
15	333	379	614

Table 2D. Estimated peak hospital occupancy rate related to mean length of stay range 8 to 15 days for 25, 30 and 50% attack rates and pandemic period 14 weeks, **with** antiviral medication

Mean length of stay, d	Attack rate, %		
	25	30	50
8	119	146	243
9	128	154	257
10	137	163	272
11	147	172	286
12	156	180	300
13	165	189	315
14	174	198	329
15	175	198	331

Table 2E. Estimated peak critical care occupancy rate by **25%** critical care admission rate, related to mean length of stay range 8 to 15 days for 25, 30 and 50% attack rates and pandemic period 14 weeks, **with** antiviral medication

Mean length of stay, d	Attack rate, %		
	25	30	50
8	30	36	61
9	32	39	64
10	34	41	68
11	37	43	71
12	39	45	75
13	41	47	79
14	44	49	82
15	44	50	83

Table 2F. Estimated peak critical care occupancy rate by **50%** critical care admission rate, related to mean length of stay range 8 to 15 days for 25, 30 and 50% attack rates and pandemic period 14 weeks, **with** antiviral medication

Mean length of stay, d	Attack rate, %		
	25	30	50
8	59	73	121
9	64	77	129
10	69	81	136
11	73	86	143
12	78	90	150
13	83	94	157
14	87	99	165
15	87	99	165

All models are based on 0.3% hospital admission rate for infected patients. Changing this rate will have a significant impact on the peak demand for hospital beds and ICU beds. The maximum number of regular hospital beds in the 15 hospitals in the three Northern provinces of the Netherlands equals 5,629 of which 3,940 could be washed out for influenza related hospital admissions (30% of all admissions is acute, non-influenza related care). The maximum number of intensive care beds which could be washed out for influenza related care equals 136.

Table 3A1. Hospital bed peak demand for different hospital admissions rates **without** antiviral medication (at day 28 after onset of the pandemic) (pandemic period 9 weeks)

	Attack rate, %					
	25		30		50	
	Mean length of stay, d					
Hospital admission rate, %	8	15	8	15	8	15
0.1	152	206	186	252	301	409
0.2	304	412	371	505	601	818
0.3	459	666	557	758	902	1,227
0.4	608	824	742	1,009	1,203	1,635
0.5	760	1,030	928	1,261	1,503	2,044
0.6	912	1,236	1,113	1,514	1,804	2,453
0.7	1,064	1,441	1,299	1,766	2,105	2,861
0.8	1,216	1,647	1,484	2,018	2,405	3,270
0.9	1,367	1,853	1,670	2,270	2,706	3,679
1.0	1,519	2,059	1,855	2,523	3,006	4,088

Table 3A2. Hospital bed peak demand for different hospital admissions rates **with** antiviral medication (at day 43 after onset of the pandemic) (pandemic period 14 weeks).

	Attack rate, %					
	25		30		50	
	Mean length of stay, d					
Hospital admission rate, %	8	15	8	15	8	15
0.1	41	56	49	66	81	111
0.2	83	112	98	133	163	221
0.3	119	175	146	198	243	331
0.4	166	225	195	266	326	443
0.5	207	281	244	332	407	553
0.6	249	337	293	398	488	664
0.7	290	393	342	465	570	775
0.8	332	449	391	531	651	885
0.9	373	505	439	598	732	996
1.0	414	562	488	664	814	1,107

In the next tables we present the **difference** (i.e. surplus or deficit) between demand and capacity for ICU beds at the peak of the pandemic for a mean length of stay of 8 and 15 days with a maximum of 136 available ICU beds for different hospital admission rates and 30% attack rate.

Table 3B1. ICU bed difference **without** antiviral medication (pandemic period 9 weeks).

	ICU admission, %					
	25		50		75	
	Mean length of stay, d					
Hospital admission rate, %	8	15	8	15	8	15
0.1	90	73	43	10	–4	–53
0.2	43	10	–50	–117	–142	–243
0.3	–3	–54	–143	–243	–282	–433
0.4	–50	–116	–235	–369	–421	–621
0.5	–96	–179	–328	–495	–560	–810
0.6	–142	–243	–421	–621	–699	–1,000
0.7	–189	–306	–514	–747	–838	–1,189
0.8	–235	–369	–606	–873	–977	–1,378
0.9	–282	–432	–699	–999	–1,117	–1,567
1.0	–328	–495	–792	–1,126	–1,255	–1,756

Table 3B2. ICU bed difference **with** antiviral medication (pandemic period 14 weeks).

	ICU admission, %					
	25		50		75	
	Mean length of stay, d					
Hospital admission rate, %	8	15	8	15	8	15
0.1	124	120	112	103	99	87
0.2	112	103	87	70	63	36
0.3	100	87	63	37	27	–13
0.4	87	70	39	3	–10	–64
0.5	75	53	14	–30	–47	–113
0.6	63	37	–11	–63	–84	–163
0.7	51	20	–35	–97	–121	–213
0.8	38	3	–60	–130	–157	–262
0.9	26	–14	–84	–163	–193	–313
1.0	14	–30	–108	–196	–230	–362

For example: with 0.3% hospital admission rate, 50% ICU admission rate and a mean length of stay of 8 days and no intervention with antiviral medication (table 3B1), a shortage of 143 ICU beds will occur at the peak of the pandemic. Dividing these 143 beds over 15 hospitals will leave every hospital with a shortage around 10 ICU beds. For a short period of time this shortage can be bridged by utilizing any form of respiratory support available in the hospitals (operating room ventilators, medical specialist and nurses, medical students etc.)

References

1. Hagenaars TJ, Van Genugten MLL, Wallinga J. Scenario analysis of transmission dynamics, health-care demand and mortality. Brief report to the Health Inspectorate of the Netherlands. 2003.

2. Van Genugten MLL, Heijnen MLA, Jager JC. Scenario ontwikkeling zorgvraag bij een influenzapandemie. RIVM rapport 217617 004. 2001.

3. Baltussen RM, Reinders A, Sprenger MJW, Postma MJ, Jager JC, Ament AJHA, et al. Estimating influenza-related hospitalization in the Netherlands. Epidemiol Infect. 1998;121:129–38.

4. Sprenger MJ, Mulder PG, Beyer WE, van Strik R, Masurel N. Impact of influenza on mortality in relation to age and underlying disease, 1967-1989. Int J Epidemiol. 1993;22:334–40.

5. Anderson TA, Hart GK, Kainer MA. Pandemic influenza-implications for critical care resources in Australia and New Zealand. J Crit Care. 2003;18:173–80.
